# Targeted faith-based and faith-placed interventions for noncommunicable disease prevention and control in low- and middle-income countries: a systematic review protocol

**DOI:** 10.1186/s13643-022-01981-w

**Published:** 2022-06-11

**Authors:** Oluwakemi Ololade Odukoya, Gursimer Jeet, Busola Adebusoye, Oluwaseun Idowu, Folasade Tolulope Ogunsola, Kolawole S. Okuyemi

**Affiliations:** 1 Department of Community Health and Primary Care, College of Medicine, University of Lagos, Lagos, Nigeria; 2 Department of Family & Preventive Medicine, University of Utah School of Medicine, 375 Chipeta Way, Suite A, Salt Lake City, UT 84108 USA; 3 School of Public Health, Post Graduate Institute of Medical Education and Research, Chandigarh, India; 4 Lifespan and Population Health Academics Unit, Clinical Sciences Building, City Hospital Campus, University of Nottingham, Nottingham, UK; 5 Department of Microbiology and Parasitology, College of Medicine, University of Lagos, Lagos, Nigeria

**Keywords:** Faith-based interventions, Faith-placed interventions, Religion and spirituality, Integrative health, Noncommunicable diseases

## Abstract

**Background:**

Low- and middle-income countries (LMICs) bear a disproportionately high burden of noncommunicable diseases (NCDs) with severe socioeconomic consequences. Targeted interventions that are faith-based or take place in faith-based settings are historically viable for health promotion and disease prevention programmes. However, evidence of their effectiveness often comes from high-income countries. This paper outlines the protocol for the systematic review of faith-based and faith-placed interventions for NCDs in low- and middle-income countries.

**Objective:**

To determine the effectiveness of faith-based and faith-placed interventions or interventions within faith-based settings targeted at NCDs and/or their risk factors in LMICs.

**Methods:**

We will conduct a systematic search of PubMed, Embase, Scopus, WHO Library, and grey literature to locate published and unpublished studies. We will consider quantitative studies that report on interventions (a) with faith-based components or that take place in faith-based settings (b) for the prevention and control of one or more of the top ten NCDs listed in the Global Burden of Disease or their known risk factors (c) occurring among adults aged 18 and above (d) that take place in one or more LMICs.

We will screen the titles, abstracts, and full text of articles for eligibility. Included articles will be critically appraised for quality and the inclusion of faith-based components by at least two independent reviewers. Data extraction will be performed for study characteristics and findings. A meta-analysis will be used to synthesize the results; if impossible, a narrative synthesis will be performed.

**Discussion:**

This review will attempt to synthesize up-to-date evidence to guide effective decision-making, allocation of health resources, and the design of future trials to test the efficacy of NCD interventions in faith-based settings. The study will increase the understanding of the existing evidence, highlight the need for additional evidence, and guide possible directions for future collaborations between public health professionals and faith-based health service providers.

**Systematic review registration:**

PROSPERO CRD42020186299

**Supplementary Information:**

The online version contains supplementary material available at 10.1186/s13643-022-01981-w.

## Key highlights

- Evidence of the effectiveness of faith-based and faith-placed interventions for preventing and controlling NCDs and their known risk factors in resource-limited settings has not been synthesized previously.
- Programme managers and policymakers can use this evidence to allocate health resources effectively considering these settings in addition to commonly used settings like schools and workplaces.
- The scope of the review covers low- and middle-income countries; there is still room for an all-encompassing review for developed countries, which can serve as a basis for comparison.

## Background

Worldwide, religious settings serve as regular contact points and media to establish social relationships within communities. They are potentially effective channels to deliver health interventions and institute behavioural change at individual and community levels [1, 2]. Faith-placed interventions have a spiritual basis and occur in organized religious settings, while faith-based interventions have a spiritual basis or are organized with the significant involvement of a faith group but do not necessarily take place in religious establishments [3–6].

Faith-based and faith-placed (FB/FP) interventions have several advantages [1, 7]. They can reach a sizeable, consistent group, provide space for programming, offer social support, and include influential leaders who can promote participation and potentially sustain programmes in the long term [8–10]. These interventions may provide a familiar setting for individuals who may feel alienated from mainstream healthcare systems due to differences in health beliefs, attitudes, or language. They can succeed in ways that traditional healthcare systems cannot [1, 2]. Furthermore, the pre-existing social networks and organizational structures of these settings tend to facilitate the adoption and maintenance of health behaviours [1, 5, 11].

There is sufficient evidence that interventions in religious settings, particularly churches, play an important role to disseminate and translate evidence-based health programmes for noncommunicable diseases [12–17]. Most LMICs are firmly embedded in religious practices as religion may serve as a psychological mechanism for coping with high levels of stress and anxiety within the suboptimal social and economic environments often prevalent in LMICs [18]. The popularity of religion in LMICs also provides an opportunity to address a large body of people. Hence, synthesizing the findings of these studies across LMICs can provide evidence for key stakeholders on the viability of such interventions and their adaptation across LMICs. Using these settings for behavioural change has been studied in several countries [1–3, 5, 13–15]. However, the evidence in LMICs, which bear a disproportionately high burden of NCD deaths, is not known.

### Theoretical framework

**Fig. 1:**
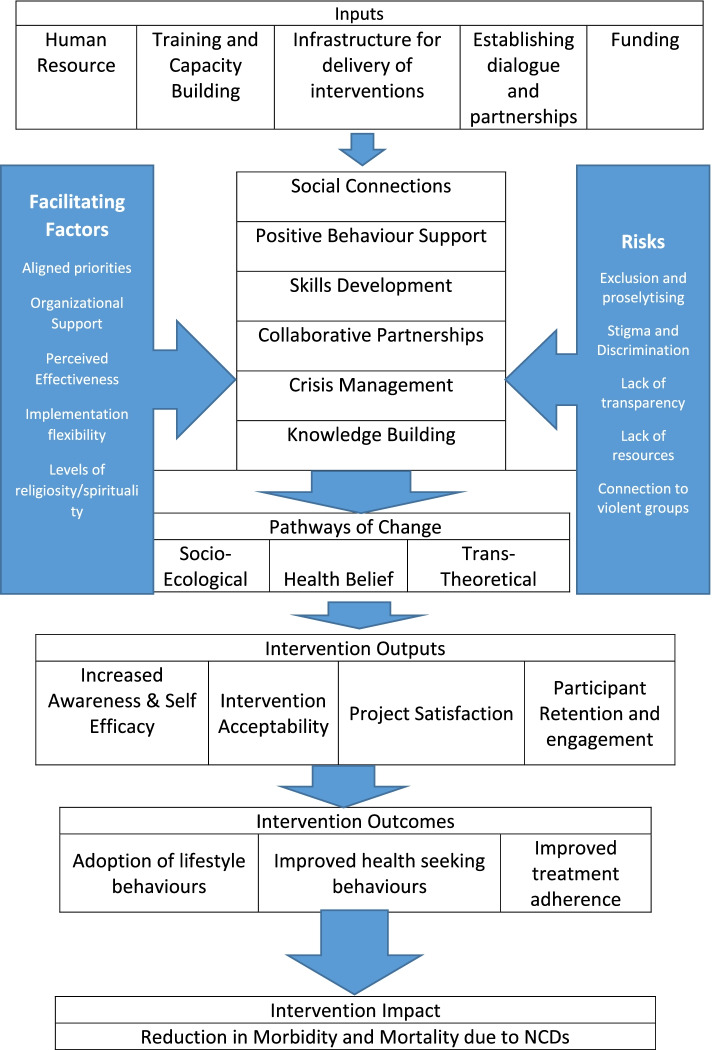
A system-based approach to evaluating faith-based ad/or faith-placed interventions For NCD’s

In a review of this nature, it is expected that there may be significant variations in the nature of the interventions to be studied. In line with the recommendations in the Cochrane Handbook of Systematic Reviews, we outline a system-based approach for evaluating faith-based and/or faith-placed interventions for NCDs. This is to highlight the causal pathways that illustrate the potential mechanisms of change of the underlying interventions and their mediators and moderators [19, 20]. Figure 1 illustrates the core elements and expected outcomes of change for the interventions. The programme inputs, such as human resources, training and capacity building, faith-based or faith-placed infrastructures, and availability of funding influence the intervention effects. Facilitators such as advocacy and the alignment of the project priorities with the faith of the beneficiaries, as well as risks such as a lack of transparency or stigma and discrimination, may influence the behavioural change pathways, the proximal and distal outputs, and ultimately the intervention outcomes and impact (Fig. 1).

## Aim

This review aims to synthesize evidence on the effectiveness of interventions targeted at NCDs and their risk factors within faith-based or faith-placed settings in LMICs. In this review, we will evaluate the various models employed in the trials as well as their theoretical underpinnings. We will assess their effectiveness using the framework outlined above, i.e. inputs, components, opportunities, risks, outputs, behaviour change pathways and how these lead to successful outcomes, and the overall impact of the interventions. Identifying the critical elements of successful interventions in faith-based settings will help elucidate the factors that could improve similar interventions’ effectiveness.

## Methods/design

The Preferred Reporting Items for Systematic reviews and meta-analyses

Protocols (PRISMA-P) checklist is used in reporting the protocol (see Additional file 1) [21].

### Eligibility criteria

#### Inclusion criteria

Studies will be included in the review if they fulfil the following criteria:

1. Studies with at least one faith-based or faith-placed component [3–6]
2. Studies with interventions targeting at least one of the top ten NCDs or their modifiable risk factors will be included. The top ten NCDs in this review will be retrieved from the list of NCDs in the 2019 Global Burden of Disease Report [22]. Because of the large number of possible risk factors for each NCD, we will limit our search to the leading NCD risk factors listed by the World Health Organization [23]. These are tobacco use, physical inactivity, harmful use of alcohol, unhealthy diets, raised blood pressure, overweight/obesity, high blood glucose, and hyperlipidemia [23]. The list of NCDs and NCD risk factors for this review are provided as a Additional file 2.
3. Studies employing the following study designs: individual or cluster randomized controlled clinical or community-based trials
4. Studies in low- and middle-income countries as defined by the World Bank in 2022 [24].
5. Articles published in any language with full English abstracts will be eligible for inclusion.

#### Exclusion criteria

Purely descriptive studies, observational studies, qualitative studies, or any non-intervention studies will be excluded. In addition, opinion pieces, letters, or any other publications lacking primary data and explicit method descriptions will also be excluded.

In cases where there are duplicate publications of the same material, the most complete and recent version will be used.

The PICO framework for the review is as follows:

### Population

Participants will be adults 18 years and older residing in LMICs. We will use the 2022 World Bank list of LMICs to identify the countries included in the review [24]. See Additional file 3)

### Intervention

For this review, the following definitions will be used.

Faith-based interventions: Interventions will be referred to as “faith-based” when they include religious or spiritual reinforcements and or are organized and operated with the significant involvement of a faith group but may or may not take place in religious settings [3–6].

Faith-placed interventions: Interventions will be referred to as faith placed if they involve religious or spiritual reinforcement and are operated with the significant involvement of a faith group and take place within religious organizations, religious congregations or houses of worship, organized religious denominations, faith-based social service agencies, and faith-based charities [3–6].

Targeted faith-based or faith-placed interventions for NCDs are described as interventions that are targeted at one or more NCDs or known risk factors for NCDs. For this review, we will limit our searches to the top ten NCDs listed to be causing the highest morbidity burden in the most recent Global Burden Disease Report. (2019) [22]. The NCDs included in this review are listed in the Additional file 3.

### Comparator

Routine health services or health programmes targeted at NCD and or their risk factors but are not faith based or faith placed.

### Outcomes

Percentage change in morbidity or mortality due to NCD, or its risk factors in the groups under comparison, or differences in the risk factor measured in the groups will serve as the primary outcomes.

Different trials may evaluate various biochemical, metabolic, or behavioural risk factors for any of the listed NCDs like high blood pressure, blood sugar, lipid profiles, physical activity, dietary intake, anthropometric measurements, tobacco, or alcohol consumption. We will abstract all the outcomes reported in trials and pool where possible. Intermediate measures such as knowledge, practice, self-efficacy, quality of life, or treatment adherence for any of the listed NCDs or their risk factors are additional variables that may be observed and will be measured as secondary outcomes. Percentage change in measurements of these will be reported.

### Information sources and search strategy

Searches of published literature will be done in the following biomedical and general reference electronic databases, without restriction to publication year or language: MEDLINE, PubMed Central; Excerpta Medica Database (EMBASE), PsycINFO, Ovid Medline, Scopus, and World Health Organization (WHO) library. In addition, clinical trials registers such as ClinicalTrials.gov and portals, trials registers of developing countries through WHO International Clinical Trials Registry Platform (ICTRP), the Pan African Clinical Trials Registry, and the Cochrane Database of Systematic Reviews will serve as additional sources of information. Also, reference lists of articles identified from the initial searches and bibliographies of systematic and nonsystematic review articles will be examined to determine relevant studies.

Authors or trial investigators may be contacted to acquire additional data related to the outcomes of interest that may be unpublished or from ongoing studies. Unpublished, completed trials and reports will also be included if identified in a database or referenced in a publication identified in the initial search.

For the preliminary search, we will identify keywords with suitable Boolean operators. Search terms will include keywords like “faith” AND “intervention” (including clinical trial/studies, randomised controlled trial) AND “NCD/Risk Factors” AND “LMICs” (Table 1)

**Table 1:** Search terms to identify the literature

PICO elements	Indexed search terms (Emtree)	Free text words (not limited to)	Search strategy
**P** (**p**atient or **p**opulation)	Noncommunicable diseases [MeSH]	(((((((((((((((((((((((((((((((((((((((((((((((((((((((((((((((Noncommunicable) OR Noncommunicable Disease) OR Noncommunicable Disease) OR Noncommunicable) OR NCD) OR Chronic disease) OR Risk Factors) OR Cancer) OR Cardiovascular disease) OR CVD) OR "CVD risk") OR hypertension) OR lifestyle factors) OR NCDs) OR Cardiovascular diseases) OR raised blood pressure) OR diabetes) OR "diabetes mellitus") OR cholesterol) OR cholestrolemia) OR cholesterolemia) OR raised cholesterol) OR high triglycerides) OR triglycerides) OR Body Mass Index) OR BMI) OR Raised BMI") OR "Raised BMI") OR Overweight) OR obesity) OR obese) OR waist circumference) OR exp alcohol drinking/) OR exp drinking/) OR tobacco) OR "tobacco smoke") OR diet*) OR nutrition) OR exp food habits) OR fruit) OR fruits) OR ("fruits and vegetables")) OR vegetables) OR vegetable consumption) OR vegetable intake) OR vegetable intake) OR low intake) OR "5 a day") OR "Five a day") OR "less than five servings") OR "physical inactivity") OR "exercise") OR " regular exercise") OR exp smoking/) OR smoking behaviour) OR "alcohol") OR alcohol consumption) OR exp stress/) OR running) OR jogging) OR walking) OR walk*) OR junk food) OR "fast food"	Non-communicable diseases [MeSH] OR (((((((((((((((((((((((((((((((((((((((((((((((((((((((((((((((Non communicable) OR Non communicable isease) OR Noncommunicable Disease) OR Noncommunicable) OR NCD) OR Chronic disease) OR Risk Factors) OR Cancer) OR Cardiovascular disease) OR CVD) OR "CVD risk") OR hypertension) OR life style factors) OR NCDs) OR Cardiovascular diseases) OR raised blood pressure) OR diabetes) OR "diabetes mellitus") OR cholesterol) OR cholestrolemia) OR cholesterolemia) OR raised cholesterol) OR high triglycerides) OR triglycerides) OR Body Mass Index) OR BMI) OR " Raised BMI") OR "Raised BMI") OR Overweight) OR obesity) OR obese) OR waist circumference) OR exp alcohol drinking/) OR exp drinking/) OR tobacco) OR "tobacco smoke") OR diet*) OR nutrition) OR exp food habits) OR fruit) OR fruits) OR ("fruits and vegetables")) OR vegetables) OR vegetable consumption) OR vegetable intake) OR vegetable intake) OR low intake) OR "5 a day") OR "Five a day") OR "less than five servings") OR "physical inactivity") OR "exercise") OR " regular exercise") OR exp smoking/) OR smoking behaviour) OR "alcohol") OR alcohol consumption) OR exp stress/) OR running) OR jogging) OR walking) OR walk*) OR junk food) OR "fast food"
**Boolean**			AND
**I** (**I**ntervention setting)	Faith (MeSH)	Faith-based organi*ation* OR faith-based organi*ation* OR religion* OR church* OR mosque OR spiritual*	Faith [MeSH] OR faith-based organi*ation* OR faith based organi*ation* OR religion* OR church* OR mosque*
**Boolean**			AND
**C** (**C**omparison)/determinants	No MeSH term used	No intervention OR routine care OR delayed intervention OR material OR print material OR education	No intervention[Text Word] OR routine care[Text Word] OR delayed intervention[Text Word] OR material[Text Word] OR print material[Text Word] OR education[Text Word]
**Boolean**			AND
**O** (primary **o**utcome)		Blood pressure OR blood sugar OR lipid profiles OR fat intake physical activity, dietary intake, fruit intake or vegetable intake or fruit and vegetable intake OR anthropometric measures OR weight OR BMI OR body mass index OR tobacco use OR alcohol consumption OR screening rate OR physical activity OR metabolic equivalents OR five servings OR treatment adherence OR utilization	(("blood pressure"[Text Word] OR "blood sugar"[Text Word] OR "lipid profiles"[Text Word] OR (("fat"[All Fields] AND ("intake"[All Fields] OR "intake s"[All Fields] OR "intakes"[All Fields]) AND ("exercise"[MeSH Terms] OR "exercise"[All Fields] OR ("physical"[All Fields] AND "activity"[All Fields]) OR "physical activity"[All Fields]) AND ("diet"[MeSH Terms] OR "diet"[All Fields] OR "dietary"[All Fields] OR "dietaries"[All Fields]) AND ("intake"[All Fields] OR "intake s"[All Fields] OR "intakes"[All Fields])) AND "fruit intake"[Text Word]) OR "vegetable intake"[Text Word] OR "fruit"[Text Word]) AND "vegetable intake"[Text Word]) OR "anthropometric measures"[Text Word] OR "weight"[Text Word] OR "BMI"[Text Word] OR "body mass index"[Text Word] OR "tobacco use"[Text Word] OR "alcohol consumption"[Text Word] OR "screening rate"[Text Word] OR "physical activity"[Text Word] OR "metabolic equivalents"[Text Word] OR "five servings"[Text Word] OR "treatment adherence"[Text Word] OR "utilization"[Text Word] OR ("kcal"[Text Word] OR "cancer control"[Text Word] OR "score"[Text Word])
**Boolean**			AND
**S** (Study design)	RCT (MeSH)	(((((((((((((((randomized controlled trial[Publication Type]) OR "randomized controlled trial"[Publication Type]) OR randomized[Text Word]) OR randomizes[Text Word]) OR random*[Text Word]) OR intervention[Text Word]) OR interventions[Text Word]) OR cluster randomization) OR cluster randomization) OR cluster randomized) OR group randomized trial) OR "group randomized") OR "group randomized") OR control) OR controlled) OR controlled trial	RCT [MeSH] OR (((((((((((((((randomized controlled trial[Publication Type]) OR "randomized controlled trial"[Publication Type]) OR randomized[Text Word]) OR randomizes[Text Word]) OR random*[Text Word]) OR intervention[Text Word]) OR interventions[Text Word]) OR cluster randomization) OR cluster randomization) OR cluster randomized) OR group randomized trial) OR "group randomized") OR "group randomized") OR control) OR controlled) OR controlled trial
**Boolean**			AND
Country	LMIC (MeSH)	“Country name” OR developing countr* OR less developed countr* OR “low-income countr	LMIC [MeSH] OR developing countr* OR less developed countr* OR “low-income countr

We will initially screen the titles and abstracts of identified manuscripts before reviewing the full texts of included papers. For papers not written in the English Language, we will use Google Translate® for translation into English and document the number of such articles in the review [25] Studies examining the effectiveness and those evaluating cost-effectiveness will be reviewed separately. The review team will consist of a community medicine expert with a background in researching the link between faith and promoting preventive behaviours for NCDs and their risk factors (OO) and a public health researcher with experience in conducting reviews of community-based interventions focused on NCD prevention and control (GJ). In addition, experts in clinical medicine, a librarian and a biostatistician will support the review.

### Data extraction and processing

Search results will be saved into Endnote files which will be de-duplicated, collated, and transferred into Rayyan for subsequent processing. Two sets of reviewers (BA, OI ) will conduct an initial independent screening of articles’ titles and abstracts using the predefined inclusion and exclusion criteria. A third reviewer will resolve disagreements (OO). Full texts of the selected articles will be obtained for further review and assessed using the same process as the title/abstract screen. Two independent reviewers (GJ, OI) will use a pretested excel-based data extraction form adapted from the Cochrane data extraction template for intervention reviews for RCT and non-RCTs to extract the data from the full texts. Information extracted will include publication characteristics such as study title, author, year, country, and study design and methodological characteristics, i.e. sample size, study population and setting, intervention type and delivery, components and of the intervention and outcome measures, loss to follow-up, and protocol publication to study fidelity of reported outcomes (see data extraction form in Additional file 4).

In addition, we will evaluate the theoretical models employed in the trials and assess inputs, components, opportunities, risks, outputs, behaviour change pathways, and how these led to successful or unsuccessful outcomes. If there are unclear or missing data related to study methods, primary outcome, or statistical parameters, the trial’s principal investigator will be contacted by email. Missing secondary outcome data will also be recorded in the data extraction form and the risk of bias tables. The results will be synthesized and presented as a descriptive summary. The inter-rater reliability for excluding studies, measured as Cohen’s kappa, will be reported [26].

### Synthesis of results

The study will be conducted and reported in line with the updated Preferred Reporting Items for Systematic Reviews and Meta-Analyses (PRISMA) statement guidelines [21]. We will conduct meta-analyses in RevMan 2012 if the included studies are sufficiently homogeneous (*I*^2^ statistic < 75%) and a minimum of two studies for any intervention being compared [27]. If there is considerable heterogeneity (*I*^2^ > 75%), we will only synthesize the results narratively. If we are unable to use RevMan, we will use STATA software v16.0 for data analysis, and we will consult a statistician to help with this process [28].Meta-analyses will be carried out separately for each outcome and the type of study design. We will use the random-effects model for all analyses to incorporate any existing heterogeneity and generate a forest plot for each comparison [28].We will carry out a narrative synthesis of the results, grouping our findings by the type of intervention and outcome measurements. We will include a table of the summary of findings for the primary outcome of this review using the Grading of Recommendations Assessment, Development and Evaluation (GRADE) approach [29].This will include the number of participants and studies for each outcome, a summary of the intervention effects, and a measure of the quality of evidence. We will classify the studies using the four levels of certainty: high, moderate, low, and very low. We will consider the following GRADE domains to evaluate the included papers, i.e. risk of bias, inconsistency, indirectness, imprecision, and publication bias [29]. The primary outcome measures will be grouped into dichotomous or continuous categories as appropriate. For dichotomous measures, we will calculate the unadjusted and adjusted risk difference or relative risk. For continuous measurements, we will calculate percentage changes from baseline, unadjusted and adjusted. These analyses will also allow us to explore heterogeneity or trials that have not accounted for clustering [28, 30]. Sample size estimates will be adjusted for design effect using an “approximation method” [28, 30]. Effective sample size will be calculated for the comparison groups by dividing the original sample size by the design effect. The design effect will be calculated as 1+ (m−1) ICC, where m is the average cluster size and ICC is the intra-cluster correlation coefficient [28, 30]. If primary data is unavailable, we will attempt to find an appropriate ICC from the literature and adjust the sample size accordingly [31]. We may undertake sensitivity analysis to identify key study parameters (sample size, trial quality, trial settings) that may affect the review findings [28].

### Methodological quality of included studies and meta bias(es) assessment

The quality of the included studies will be assessed using the Hamilton Effective Public Health Practice Project Checklist for quantitative studies [32].This tool uses eight domains to evaluate the quality of studies, i.e. selection bias, study design, confounders, blinding, data collection practices, analysis, invention integrity, and withdrawals and dropouts. Each study will be assessed as strong, moderate, or weak in each of these domains. Two investigators (BA, OI ) will independently assess the quality of included studies, and a third reviewer (OO) will resolve, if any. A study would be rated as strong if it has no weak ratings, moderate if it has only 1 weak rating, and weak if it has 2 or more weak ratings [32]. Besides, we will assess the degree to which each faith-based component is integrated into the intervention. This will be done using a faith-based integration tool (FIAT), which quantifies faith-health integration [33]. For an outcome where more than ten trials are available, the likelihood of reporting bias will be explored. Funnel plots will be created to visually assess sources of asymmetry, such as small-study effects or publication bias. If small-study effects are found to result in asymmetry, then further sensitivity analysis will be undertaken to show its effects on the pooled results [28, 32].

## Discussion

The scope of the review covers low- and medium-income countries where innovative and cost-effective ways are needed to curtail the rising double burden of NCDs and infectious diseases. Religion and/or spirituality is known to shape individual or communal beliefs and behaviours [34–37]. Addressing NCD interventions through the lens of religion or spirituality may serve as an innovative way of health promotion in LMICs. If proven effective, these interventions may be sustainable and cost-effective in resource-poor settings. Settings-based approaches have been studied widely for NCDs [38, 39]; however, the evidence may vary depending on the setting under purview. Some researchers have demonstrated the success of religious beliefs in promoting behavioural changes that reduce the NCD burden [5, 17, 40–43]. For instance, religious belief systems are known to maintain low smoking rates [44, 45], reduce harmful drinking [46], and overeating [1]. Similar to developed nations, the dynamics in developing country settings are driven by religious and political agendas rather than socio-economic issues alone [47, 48]. Studies evaluating the effectiveness of faith-based and or faith-placed interventions for health promotion have pointed towards a positive relation [12–15]. However, the quality of evidence was low in reviews conducted in 2011–2012. The evidence is scarce for NCDs interventions in particular [1, 8, 41].

## Supplementary Information

**Additional file 1.** PRISMA-P checklist. The Preferred Reporting Items for Systematic reviews and Meta-Analyses Protocols (PRISMA-P) checklist is used in reporting the protocol

**Additional file 2.** The list of the Non-Communicable Diseases and risk factors to be included in the review. Review The top ten NCDs in the 2019 Global Burden of Disease Report and the leading NCD risk factors- World Health Organisation.

**Additional file 3.** The list of the low and middle income countries to be included in the review. The 2022 World Bank list of low and middle income countries.

**Additional file 4.** Data extraction form.

## Abbreviations

FB: Faith based
FP: Faith placed
LMICs: Low- and middle-income countries
NCDs: Noncommunicable diseases
WHO: World Health Organization
PROSPERO: Prospective Register of Systematic Reviews
EMBASE: Excerpta Medica Database
PRISMA-P: Preferred Reporting Items for Systematic reviews and Meta-Analyses Protocols
PICO: Population, intervention, comparator, and outcome
ICTRP: International Clinical Trials Registry Platform
FIAT: Faith-based integration tool
