# Supplementary material for: Targeted faith-based and faith-placed interventions for noncommunicable disease prevention and control in low- and middle-income countries: a systematic review protocol
Source: Syst Rev. 2022 Jun 11;11:119. doi: 10.1186/s13643-022-01981-w (PMC9188080; doi:10.1186/s13643-022-01981-w)
Supplement: Supplementary file 2 — Additional file 2. The list of the Non-Communicable Diseases and risk factors to be included in the review. Review The top ten NCDs in the 2019 Global Burden of Disease Report and the leading NCD risk factors- World Health Organisation. [file 13643_2022_1981_MOESM2_ESM.docx]

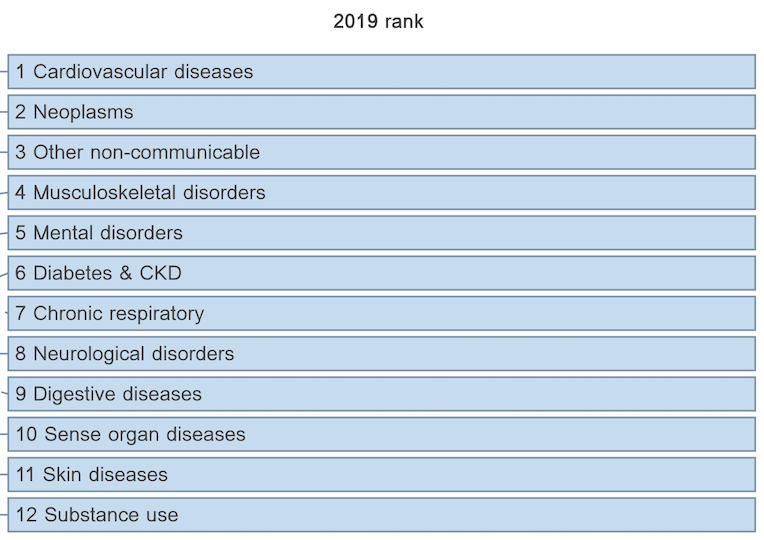


| **List of NCD risk factors to be included in the review** |
| --- |
| Tobacco use |
| Physical Inactivity |
| Harmful use of alcohol |
| Unhealthy diet |
| Raised blood pressure |
| Overweight/Obesity |
| High blood glucose |
| Hyperlipidemia |
