# Supplementary material for: Targeted faith-based and faith-placed interventions for noncommunicable disease prevention and control in low- and middle-income countries: a systematic review protocol
Source: Syst Rev. 2022 Jun 11;11:119. doi: 10.1186/s13643-022-01981-w (PMC9188080; doi:10.1186/s13643-022-01981-w)
Supplement: Supplementary file 3 — Additional file 3. The list of the low and middle income countries to be included in the review. The 2022 World Bank list of low and middle income countries. [file 13643_2022_1981_MOESM3_ESM.docx]

|  | Angola |
| --- | --- |
|  | Algeria |
|  | Bangladesh |
|  | Belize |
|  | Benin |
|  | Bhutan |
|  | Bolivia |
|  | Cabo Verde |
|  | Cambodia |
|  | Cameroon |
|  | Comoros |
|  | Congo, Rep. |
|  | Côte d'Ivoire |
|  | Djibouti |
|  | Egypt, Arab Rep. |
|  | El Salvador |
|  | Eswatini |
|  | Ghana |
|  | Haiti |
|  | Honduras |
|  | India |
|  | Indonesia |
|  | Iran, Islamic Rep |
|  | Kenya |
|  | Kiribati |
|  | Kyrgyz Republic |
|  | Lao PDR |
|  | Lesotho |
|  | Mauritania |
|  | Micronesia, Fed. Sts. |
|  | Mongolia |
|  | Morocco |
|  | Myanmar |
|  | Nepal |
|  | Nicaragua |
|  | Nigeria |
|  | Pakistan |
|  | Papua New Guinea |
|  | Philippines |
|  | Samoa |
|  | São Tomé and Principe |
|  | Senegal |
|  | Solomon Islands |
|  | Sri Lanka |
|  | Tanzania |
|  | Tajikistan |
|  | Timor-Leste |
|  | Tunisia |
|  | Ukraine |
|  | Uzbekistan |
|  | Vanuatu |
|  | Vietnam |
|  | West Bank and Gaza |
|  | Zambia |
|  | Zimbabwe |
