# Supplementary material for: Targeted faith-based and faith-placed interventions for noncommunicable disease prevention and control in low- and middle-income countries: a systematic review protocol
Source: Syst Rev. 2022 Jun 11;11:119. doi: 10.1186/s13643-022-01981-w (PMC9188080; doi:10.1186/s13643-022-01981-w)
Supplement: Supplementary file 4 — Additional file 4. Data extraction form. [file 13643_2022_1981_MOESM4_ESM.doc]

#
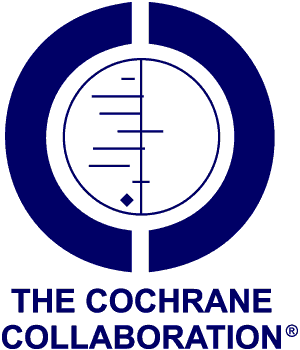
Data collection form

Intervention review – RCTs and non-RCTs

| **Review title or ID** |
| --- |
|  |

| **Study ID** *(surname of first author and year first full report of study was published e.g. Smith 2001)* |
| --- |
|  |

| **Report IDs of other reports of this study** *(e.g. duplicate publications, follow-up studies)* |
| --- |
|  |

| **Notes:** |
| --- |

## General Information

| 1. Date form completed *(dd/mm/yyyy)* | |  |
| --- | --- | --- |
| 1. Name/ID of person extracting data | |  |
| 1. Report title   *(title of paper/ abstract/ report that data are extracted from)* | |  |
| 1. Report ID   *(if there are multiple reports of this study)* | |  |
| 1. Reference details | |  |
| 1. Report author contact details | |  |
| 1. Publication type   *(e.g. full report, abstract, letter)* | |  |
| 1. Study funding source   *(including role of funders)* | |  |
| **Possible conflicts of interest**  *(for study authors)* | |  |
| 1. Notes: |  | |

## Eligibility

| **Study Characteristics** | | | **Review Inclusion Criteria**  *(Insert inclusion criteria for each characteristic as defined in the Protocol)* | **Yes/ No / Unclear** | **Location in text**  *(pg & ¶/fig/table)* |
| --- | --- | --- | --- | --- | --- |
| 1. Type of study | | | Randomised trial |  |  |
|  |  |  | Non-randomised trial |  |  |
|  |  |  | Controlled before-after study   - Contemporaneous data collection - At least 2 intervention and 2 control clusters |  |  |
|  |  |  | Interrupted time series OR  Repeated measures study   - At least 3 timepoints before and 3 after the intervention - Clearly defined intervention point |  |  |
|  |  |  | Other design (specify): |  |  |
| 1. Participants | | |  |  |  |
| 1. Types of intervention | | |  |  |  |
| 1. Types of outcome measures | | |  |  |  |
| 1. Decision: | |  | | | |
| 1. Reason for exclusion | | |  | | |
| 1. Notes: |  | | | | |

**DO NOT PROCEED IF STUDY EXCLUDED FROM REVIEW**

## Population and setting

|  | | **Description**  *Include comparative information for each group (i.e. intervention and controls) if available* | **Location in text**  *(pg & ¶/fig/table)* |
| --- | --- | --- | --- |
| 1. Population description   *(from which study participants are drawn)* | |  |  |
| 1. Setting   *(including location and social context)* | |  |  |
| 1. Inclusion criteria | |  |  |
| 1. Exclusion criteria | |  |  |
| 1. Method/s of recruitment of participants | |  |  |
| 1. Notes: |  | | |

## Methods

|  | | **Descriptions as stated in report/paper** | **Location in text**  *(pg & ¶/fig/table)* |
| --- | --- | --- | --- |
| 1. Aim of study | |  |  |
| 1. Study Design | |  |  |
| 1. Unit of allocation   *(by individuals, clusters)* | |  |  |
| 1. Start date | |  |  |
| 1. End date | |  |  |
| 1. Duration of participation   *(from recruitment to last follow-up)* | |  |  |
| 1. Notes: |  | | |

## Risk of Bias assessment

| **Domain** | | **Risk of bias**  *Low/ High/Unclear* | **Support for judgement** | **Location in text**  *(pg & ¶/fig/table)* |
| --- | --- | --- | --- | --- |
| 1. Random sequence generation   *(selection bias)* | |  |  |  |
| 1. Allocation concealment   *(selection bias)* | |  |  |  |
| 1. Blinding of participants and personnel   *(performance bias)* | |  | **Outcome group: All/** |  |
| *(if required)* | |  | **Outcome group:** |  |
| 1. Blinding of outcome assessment   *(detection bias)* | |  | **Outcome group: All/** |  |
| *(if required)* | |  | **Outcome group:** |  |
| 1. Incomplete outcome data   *(attrition bias)* | |  |  |  |
| 1. Selective outcome reporting?   *(reporting bias)* | |  |  |  |
| 1. Other bias | |  |  |  |
| 1. Notes: |  | | | |

## Participants

|  | | **Description as stated in report/paper** | **Location in text**  (pg & ¶/fig/table) |
| --- | --- | --- | --- |
| 1. Total no. randomised   *(or total pop. at start of study for NRCTs)* | |  |  |
| 1. Clusters   *(if applicable, no., type, no. people per cluster)* | |  |  |
| 1. Baseline imbalances | |  |  |
| 1. Withdrawals and exclusions   *(if not provided below by outcome)* | |  |  |
| 1. Age | |  |  |
| 1. Sex | |  |  |
| 1. Race/Ethnicity | |  |  |
| 1. Severity of illness | |  |  |
| 1. Co-morbidities | |  |  |
| 1. Other treatment received   *(additional to study intervention)* | |  |  |
| 1. Other relevant sociodemographics | |  |  |
| 1. Subgroups measured | |  |  |
| 1. Subgroups reported | |  |  |
| 1. Notes: |  | | |

## Intervention groups

*Copy and paste table for each intervention and comparison group*

### Intervention Group 1

|  | | **Description as stated in report/paper** | **Location in text**  *(pg & ¶/fig/table)* |
| --- | --- | --- | --- |
| 1. Group name | |  |  |
| 1. No. randomised to group   *(specify whether no. people or clusters)* | |  |  |
| 1. Description   *(include sufficient detail for replication, e.g. content, dose, components; if it is a natural experiment, describe the pre-intervention)* | |  |  |
| 1. Duration of treatment period | |  |  |
| 1. Timing   *(e.g. frequency, duration of each episode)* | |  |  |
| 1. Delivery   *(e.g. mechanism, medium, intensity, fidelity)* | |  |  |
| 1. Providers   *(e.g. no., profession, training, ethnicity etc. if relevant)* | |  |  |
| 1. Co-interventions | |  |  |
| 1. Economic variables   *(i.e. intervention cost, changes in other costs as result of intervention)* | |  |  |
| 1. Resource requirements to replicate intervention   *(e.g. staff numbers, cold chain, equipment)* | |  |  |
| 1. Notes: |  | | |

## Outcomes

### Outcome 1

|  | | **Description as stated in report/paper** | | **Location in text**  *(pg & ¶/fig/table)* |
| --- | --- | --- | --- | --- |
| 1. Outcome name | |  | |  |
| 1. Time points measured   *(specify whether from start or end of intervention)* | |  | |  |
| 1. Time points reported | |  | |  |
| 1. Outcome definition   *(with diagnostic criteria if relevant and note whether the outcome is desirable or undesirable if this is not obvious)* | |  | |  |
| 1. Person measuring/ reporting | |  | |  |
| 1. Unit of measurement   *(if relevant)* | |  | |  |
| 1. Scales: upper and lower limits   *(indicate whether high or low score is good)* | |  | |  |
| 1. Is outcome/tool validated? | | *Yes/No/Unclear* |  |  |
| 1. Imputation of missing data   *(e.g. assumptions made for ITT analysis)* | |  | |  |
| 1. Assumed risk estimate   *(e.g. baseline or population risk noted in Background)* | |  | |  |
| 1. Notes: |  | | | |

## Results

### For randomised or non-randomised trial - Dichotomous outcome

|  | | **Description as stated in report/paper** | | | | | **Location in text**  *(pg & ¶/fig/table)* |
| --- | --- | --- | --- | --- | --- | --- | --- |
| 1. Comparison | |  | | | | |  |
| 1. Outcome | |  | | | | |  |
| 1. Subgroup | |  | | | | |  |
| 1. Time point   *(specify whether from start or end of intervention)* | |  | | | | |  |
| 1. Results   *Note whether:*  *post-intervention OR*  *change from baseline*  *And whether*  *Adjusted OR*  *Unadjusted* | | **Intervention** | | | **Comparison** | |  |
|  |  | No. events | No. participants | | No. events | No. participants |  |
|  |  |  |  |  |  |  |  |
| 1. Baseline data | | **Intervention** | | | **Comparison** | |  |
|  |  | No. events | No. participants | | No. events | No. participants |  |
|  |  |  |  |  |  |  |  |
| 1. No. missing participants and reasons | |  | | |  | |  |
| 1. No. participants moved from other group and reasons | |  | | |  | |  |
| 1. Any other results reported | |  | | | | |  |
| 1. Unit of analysis   *(e.g. by individuals, health professional, practice, hospital, community)* | |  | | | | |  |
| 1. Statistical methods used and appropriateness of these methods   *(e.g. adjustment for correlation)* | |  | | | | |  |
| 1. Reanalysis required?   *(if yes, specify why, e.g. correlation adjustment)* | | *Yes/No/Unclear* | |  | | |  |
| 1. Reanalysis possible? | | *Yes/No/Unclear* | |  | | |  |
| 1. Reanalysed results | |  | | | | |  |
| 1. Notes: |  | | | | | | |

### For randomised or non-randomised trial - Continuous outcome

|  | | | **Description as stated in report/paper** | | | | | | | | **Location in text**  *(pg & ¶/fig/table)* | |
| --- | --- | --- | --- | --- | --- | --- | --- | --- | --- | --- | --- | --- |
| 1. Comparison | | |  | | | | | | | |  | |
| 1. Outcome | | |  | | | | | | | |  | |
| 1. Subgroup | | |  | | | | | | | |  | |
| 1. Time point   *(specify whether from start or end of intervention)* | | |  | | | | | | | |  | |
| 1. Post-intervention or change from baseline? | | |  | | | | | | | |  | |
| 1. Results   *Note whether:*  *post-intervention OR*  *change from baseline*  *And whether*  *Adjusted OR*  *Unadjusted* | | **Intervention** | | | | | | **Comparison** | | |  |  |
|  |  | Mean | | SD (or other variance) | No. participants | | | Mean | SD (or other variance) | No. participants |  |  |
|  |  |  |  |  |  |  |  |  |  |  |  |  |
| 1. Baseline data | | **Intervention** | | | | | | **Comparison** | | |  |  |
|  |  | Mean | | SD (or other variance) | No. participants | | | Mean | SD (or other variance) | No. participants |  |  |
|  |  |  |  |  |  |  |  |  |  |  |  |  |
| 1. No. missing participants and reasons | | |  | | | |  | | | |  | |
| 1. No. participants moved from other group and reasons | | |  | | | |  | | | |  | |
| 1. Any other results reported | | |  | | | | | | | |  | |
| 1. Unit of analysis   *(e.g. by individuals, health professional, practice, hospital, community)* | | |  | | | | | | | |  | |
| 1. Statistical methods used and appropriateness of these methods   *(e.g. adjustment for correlation)* | | |  | | | | | | | |  | |
| 1. Reanalysis required?   *(if yes, specify why)* | | | *Yes/No/Unclear* | | |  | | | | |  | |
| 1. Reanalysis possible? | | | *Yes/No/Unclear* | | |  | | | | |  | |
| 1. Reanalysed results | | |  | | | | | | | |  | |
| 1. Notes: |  | | | | | | | | | | | |

### For randomised or non-randomised trial - Other outcome

|  | | **Description as stated in report/paper** | | | | | **Location in text**  *(pg & ¶/fig/table)* |  |
| --- | --- | --- | --- | --- | --- | --- | --- | --- |
| 1. Comparison | |  | | | | |  |  |
| 1. Outcome | |  | | | | |  |  |
| 1. Subgroup | |  | | | | |  |  |
| 1. Time point   *(specify whether from start or end of intervention)* | |  | | | | |  |  |
| 1. Type of outcome | |  | | | | |  |  |
| 1. Results | | Intervention result | SD (or other variance) | | Control result | SD (or other variance) |  |  |
|  |  |  |  |  |  |  |  |  |
|  |  | Overall results | | | SE (or other variance) | |  |  |
|  |  |  |  |  |  |  |  |  |
| 1. No. participant | | Intervention | | | Control | |  |  |
|  |  |  |  |  |  |  |  |  |
| 1. No. missing participants and reasons | |  | | |  | |  | |
| 1. No. participants moved from other group and reasons | |  | | |  | |  | |
| 1. Any other results reported | |  | | | | |  | |
| 1. Unit of analysis   *(e.g. by individuals, health professional, practice, hospital, community)* | |  | | | | |  | |
| 1. Statistical methods used and appropriateness of these methods | |  | | | | |  | |
| 1. Reanalysis required?   *(if yes, specify why)* | |  | |  | | |  | |
| 1. Reanalysis possible? | |  | |  | | |  | |
| 1. Reanalysed results | |  | | | | |  | |
| 1. Notes: |  | | | | | | | |

***For controlled before-after study***

|  | | **Description as stated in report/paper** | | | | | **Location in text**  *(pg & ¶/fig/table)* |  | |
| --- | --- | --- | --- | --- | --- | --- | --- | --- | --- |
| 1. Comparison | |  | | | | |  |  | |
| 1. Outcome | |  | | | | |  |  | |
| 1. Subgroup | |  | | | | |  |  | |
| 1. Timepoint   *(specify whether from start or end of intervention)* | |  | | | | |  |  | |
| 1. Post-intervention or change from baseline? | |  | | | | |  | |  |
| 1. Results | | Intervention result | SD (or other variance) | | Control result | SD (or other variance) |  |  | |
|  |  |  |  |  |  |  |  |  |  |
|  |  | Overall results | | | SE (or other variance) | |  |  | |
|  |  |  |  |  |  |  |  |  |  |
| 1. No. participants | | Intervention | | | Control | |  |  | |
|  |  |  |  |  |  |  |  |  |  |
| 1. No. missing participants and reasons | |  | | |  | |  |  | |
| 1. No. participants moved from other group and reasons | |  | | |  | |  |  | |
| 1. Any other results reported | |  | | | | |  |  | |
| 1. Unit of analysis   *(individuals, cluster/ groups or body parts)* | |  | | | | |  |  | |
| 1. Statistical methods used and appropriateness of these methods | |  | | | | |  |  | |
| 1. Reanalysis required?   *(specify)* | | *Yes/No/Unclear* | |  | | |  |  | |
| 1. Reanalysis possible? | | *Yes/No/Unclear* | |  | | |  |  | |
| 1. Reanalysed results | |  | | | | |  |  | |
| 1. Notes: |  | | | | | | | | |

### For interrupted time series or repeated measures study

|  | | **Description as stated in report/paper** | | | | | | **Location in text**  *(pg & ¶/fig/table)* |  |
| --- | --- | --- | --- | --- | --- | --- | --- | --- | --- |
| 1. Comparison | |  | | | | | |  |  |
| 1. Outcome | |  | | | | | |  |  |
| 1. Subgroup | |  | | | | | |  |  |
| 1. Length of timepoints measured   *(e.g. days, months)* | |  | | | | | |  |  |
| **Total period measured** | |  | | | | | |  |  |
| 1. No. participants measured | |  | | | | | |  |  |
| 1. No. missing participants and reasons | |  | | | | | |  |  |
| 1. No. timepoints measured | | 1. Pre-intervention | | | | 1. Post-intervention | |  |  |
|  | | | |  | |  |  |
| 1. Mean value   *(with variance measure)* | |  | | | |  | |  |  |
| 1. Difference in means (post – pre) | |  | | | | | |  |  |
| 1. Percent relative change | |  | | | | | |  |  |
| 1. Result reported by authors   *(with variance measure)* | |  | | | | | |  |  |
| 1. Unit of analysis   *(individuals or cluster/ groups)* | |  | | | | | |  |  |
| 1. Statistical methods used and appropriateness of these methods | |  | | | | | |  |  |
| 1. Reanalysis required?   *(specify)* | | *Yes/No/Unclear* | | |  | | |  |  |
| 1. Reanalysis possible? | | *Yes/No/Unclear* | | |  | | |  |  |
| 1. Individual timepoint results | |  | | | | | |  |  |
| 1. Read from figure? | | *Yes/No/Unclear* | |  | | | |  |  |
| 1. Reanalysed results | | **Change in level** | **SE** | | | **Change in slope** | **SE** |  |  |
|  |  | | |  |  |  |
| 1. Notes: |  | | | | | | | | |

## Applicability

| 1. Have important populations been excluded from the study?   *(consider disadvantaged populations, and possible differences in the intervention effect)* | | *Yes/No/Unclear* |  |  |
| --- | --- | --- | --- | --- |
| 1. Is the intervention likely to be aimed at disadvantaged groups?   *(e.g. lower socioeconomic groups)* | | *Yes/No/Unclear* |  |  |
| 1. Does the study directly address the review question?   *(any issues of partial or indirect applicability)* | | *Yes/No/Unclear* |  |  |
| 1. Notes: |  | | | |

## Other information

|  | | **Description as stated in report/paper** | **Location in text**  *(pg & ¶/fig/table)* |  |
| --- | --- | --- | --- | --- |
| 1. Key conclusions of study authors | |  |  |  |
| 1. References to other relevant studies | |  |  |  |
| 1. Correspondence required for further study information   *(what and from whom)* | |  | |  |
| 1. Further study information requested   *(from whom, what and when)* | |  | |  |
| 1. Correspondence received   *(from whom, what and when)* | |  | |  |
| 1. Notes: |  | | | |
